# Rush Medical College

**Published:** 1869-10

**Authors:** 


### *Rush Medical College.*

The opening exercises of the Twenty-seventh Annual Session occurred Wednesday evening, Sept. 29th. Introductory Lecture by Prof. E. L. Holmes. An unusually large number of students attended. Further particulars will be given the ensuing month, as our manuscript report has been unfortunately mislaid.

